# Supplementary material for: Individual Resilience Interventions: A Systematic Review in Adult Population Samples over the Last Decade
Source: Int J Environ Res Public Health. 2021 Jul 16;18(14):7564. doi: 10.3390/ijerph18147564 (PMC8307772; doi:10.3390/ijerph18147564)
Supplement: Supplementary file 1 [file ijerph-18-07564-s001.zip › ijerph-1258319-supplementary/Supplementary material S1_Rationale for exclusion.pdf]

Supplementary Material S1. Rationale for exclusion

Author, year	Reasons for exclusion						
	Not a intervention	Intervention not targeting psychological resilience as an objective, aim, purpose or hypothesis.	Intervention not targeting individual resilience	No valid resilience scale was used as an outcome measure	Not a CT or a NRCS	Protocol - Intervention study not found or date of publication out of range (2021)	Full text not available
Aboody, Siev & Doron (2020)				X			
Alschuler et al. (2018)							X
Barry et al. (2019)							X
Belleville et al. (2019)							X
Bernburg, Groneberg & Mache (2019)				X			
Bright et al. (2019)		X					
Carrico et all. (2016)		X					
Castro et al. (2019)		X					
Chesak et al. (2019)					X		
Chesak et al. (2020)							X
Chin et al. (2019)		X					
Church, Sparks & Clond (2016)		X					
Cleary et al. (2018)	X						
Crane et al. (2018)				X			
de Vibe et al. (2018)		X					

Deforche et al. (2018)			X				
Ehde et al. (2015)		X					
Fancourt et al. (2016)		X					
Fauerbach et al. (2020)				X			
Flett et al. (2019)							X
Funes et al. (2019)		X					
Galante et al. (2018)				X			
Games, Thompson & Barret (2020)							X
Geschwind et al. (2011)		X					
Gilmartin et al. (2017)	X						
Glass et al. (2019)					X		
Greenberg et al. (2019)							X
Guest et al. (2016)		X					
Guest etal. (2015)							X
Hahm et al. (2019)		X					
Harrison et al. (2019)			X				
Herrero et al. (2019)						X	
Highland et al. (2018)		X					
Hirani et al. (2018)							X

Hoorelbeke & Koster (2017)		X				
Ikai et al. (2014)						X
Im et al. (2016)						X
Ínci & Temel (2016)			X			
Isaacs et al. (2017)	X					
Jennings et al. (2013)		X				
Jennings et al. (2019)		X				
Jiang et al. (2019)						X
Jones et al. (2019)			X			
Jonhson et al. (2014)			X			
Jonhson et al. (2015)			X			
Joyce et al. (2019)				X		
Juul et al. (2020)		X				
Kasser & Zia (2020)	X					
Kent et al. (2011)						X
Kingston et al. (2014)			X			
Kinser et al. (2016)			X			
Köhle et al. (2015)					X	
Kreutzer et al. (2018)						X
Lai et al. (2020)					X	

Lebares et al. (2018)			X		
Letwin & Silverman (2017)			X		
Litvin et al. (2020)			X		
Luthar et al. (2017)			X		
Mache et al. (2016)					X
Mache et al. (2017)					X
McCann, Songprakun & Stephenson (2017)					X
Milicevic, Milton & O'Loughlin (2016)			X		
Mistretta et al. (2018)		X			
Monstross-Thomas et al. (2015)				X	
Moorfield & Cope (2020)	X				
Palma-Goméz et al. (2020)				X	
Park et al. (2020)					X
Pérez-de la Cruz (2020)		X			
Peter & Tran (2018), Austria			X		
Petree, Broome &					X

Bennett (2012)						
Pyatak et al. (2018)		**X**				
Reyes et al. (2020)				**X**		
Roghanchi et al. (2013)						**X**
Rose et al. (2013)			**X**			
Roy, Highland & Costanzo (2015)			**X**			
Schure et al. (2019)						**X**
Shim et al. (2017)	**X**					
Skeffington et al. (2016)		**X**				
Smeets et al. (2014)			**X**			
Smith et al. (2019)						**X**
Songprakun & McCann (2015)	**X**					
Steinberg, Klatt & Duchemin (2016)						**X**
Sylvia et al. (2020)				**X**		
Tobe, Sakka & Kamibeppu (2019)					**X**	
Trent et al. (2019)		**X**				
Trombka et al. (2018)					**X**	

Üzar-Özçetin & Hiçdurmaz (2019)							**X**
van der Stouwe et al. (2016)						**X**	
Varker & Devilly (2012)				**X**			
Victor, Teismann & Willutzki (2017)							**X**
Vranceanu et al. (2016)				**X**			
Wang, Nan & Zhang (2017)				**X**			
Weiner et al. (2020)						**X**	
Weiss et al. (2017)				**X**			
Yuan et al. (2014)				**X**			
Zale et al. (2018)				**X**			
Zamirinejad et al. (2014)							**X**
Zurita-Ortega et al. (2018)					**X**		
**Total excluded by criteria**	**7**	**22**	**2**	**26**	**6**	**9**	**26**
