# Supplementary material for: Individual Resilience Interventions: A Systematic Review in Adult Population Samples over the Last Decade
Source: Int J Environ Res Public Health. 2021 Jul 16;18(14):7564. doi: 10.3390/ijerph18147564 (PMC8307772; doi:10.3390/ijerph18147564)
Supplement: Supplementary file 1 [file ijerph-18-07564-s001.zip › ijerph-1258319-supplementary/Supplementary material S2_Detailed summary of the studiesí» characteristics.pdf]

Author, year of publication and setting	Study Design	Moment of intervention in relation to stress exposure	Population	Participants (number of randomized and demographics)<br>Intervention arm(s)	Control arm	Study objective /hypothesis	Theoretical frame	Intervention description (duration of program, duration of sessions, type of delivery)<br>Intervention arm	Control arm	Measurement time points	Outcome measure(s)<br>Resilience	Mental Health related	Stress related	Efectiveness	Methodological quality
Aikens et al. (2014), USA	RCT	During exposure	Employees	N=44	N=45	Hypothesis: a shortened, Web-based workplace mindfulness program would (1) increase measures of mindfulness, (2) decrease stress, (3) enhance resiliency, and (4) improve employee vigor and work engagement, thereby resulting in an increase in positive organizational behavior and enhanced employee well-being.	Mindfulness -Based Stress Reduction (MBSR)	**Description:** Participants were given access to their unique training dashboard through the program Web site and also received a workbook, which corresponded to each weekly lesson and contained a practice guide. Mental fitness and focusing techniques: (1) seated focus exercises on the breath, physical sensation, sound, and thought; (2) body scan; (3) walking meditation; (4) movement exercise with focus on various stretching postures and awareness of sensation and (5) 3-minute breathing pause; performance-oriented skills such as successful handling of stressful situations, recognition of autopilot and automatic mind scripts, mindful communication, presentation preparedness, and mindful problem solving.<br>**Duration of the program:** 7 weeks<br>**Duration of the sessions:** weekly hour-long virtual class meetings<br>**Tipe of delivery:** online, with 1 introductory in-person meeting	Wait-list control group	T1 - baseline<br>T2 - post-intervention<br>T3 - 6 month follow up	CD-RISC 25	No	Yes	Yes	17 (fair)
Burton, Qeadan & Burge (2019); USA	NRCS	During exposure	Veterans with PTSD	N=10<br>Male - 80%<br>Female - 20%<br>Mean age - 48; SD - 15	N=10<br>Male - 80%<br>Female - 20%<br>Mean age - 46; SD - 13	Aim: explore the effects of EAP on PTSD symptoms<br>Hypothesis: veterans with PTSD who participate in a standardized program would experience decreased PTSD symptoms and would demonstrate increased resilience as compared with individuals who do not receive EAP intervention.	Equine-assisted psychotherapy (EAP)	**Description:** An EAGALA-certified and -licensed occupational therapist performed the therapy, along with a professional horse handler who was present for safety purposes. Effective and deliberate techniques were utilized where the horses were metaphors in specific ground-based experiences. Metaphors work by allowing participants to perform an individualized transderivational search, a psychological process that facilitates locating the area of meaning most resonant with their current psychological challenges and state of functioning. They manteined the on-going standard PTSD therapy.<br>**Duration of the program:** 6 weeks<br>**Duration of the sessions:** weekly 1-hour session<br>**Tipe of delivery:** in-person group sessions	Usual care control group<br>**Description:** on-going standard PTSD therapy	T1 - baseline<br>T2 - post-intervention	CD-RISC 25	Yes	No	No	17 (fair)
Champion, Economides & Chandler (2018), UK and USA	RCT	Pre-exposure	General population (convenience and snowball sampling)	N=38<br>Male - 55.2%<br>Female - 44.8%<br>Mean age - 40.17; SD - 4.08	N=36<br>Male - 27.3%<br>Female - 72.7%<br>Mean age - 38.21; SD - 6.75	Aim: analyse the impact of engaging with the self-guided mindfulness meditation (MM) app "Headspace" in aspects like satisfaction with life, perceived stress and resilience in the general population.	Mindfulness Meditation (MM)	**Description:** Participants were sent a code which provided 30-days of free access to the MM app,and instructions on how to download the app and redeem their code. The program consists of three levels “Foundations 1–3”, with each level comprising 10 sessions (30 in total). The program is intended to introduce the key principles behind mindfulness and how to apply mindfulness to their daily life, using techniques such as breathawareness, body scanning, and noting.<br>**Duration of the program:** 30 days<br>**Duration of the sessions:** sessions begin with a duration of 10 minutes, though users have the option to increase the duration to 15 and 20 minutes during levels 2 and 3 respectively.<br>**Tipe of delivery:** self-administered through an app	Wait-list control group	T1 - baseline<br>T2 - during<br>T3 - post-intervention	Resilience Scale	Yes	Yes	Yes	18 (fair)
Christopher et al. (2018), USA	RCT	During exposure	Law enforcement officers (LEO's)	N=24<br>Male - 10%<br>Female - 90%<br>Mean age - 44.73; SD - 6.63	N=25<br>Male - 10%<br>Female - 90%<br>Mean age - 43.22; SD - 5.43	Aim: assess feasibility and gather preliminary outcome data on MBRT for law enforcemente officers, hypothesizing that participants in the active arm would evidence improved psychological health and risk outcomes (including resilience), reduced agression and anger and improved regulation of stress reactivity.	Mindfulness-Based Resilience Training (Kabat-Zinn, 1990)	**Description:** Experiential and didactic exercises, including body scan, sitting and walking meditations, mindful movement, and group discussion. Content and language were adapted for an LEO population; the primary focus of the curriculum was learning strategies to manage stressors inherent to police work, including critical incidents, job dissatisfaction, and public scrutiny, as well as interpersonal, affective and behavioral challenges common to LEOs’ lives. To supplement in-session content and support practice between sessions, MBRT participants were each given an iPod Touch programmed with guided practices and monitoring software (iMINDr).<br>**Duration of the program:** 8 weeks<br>**Duration of the sessions:** weekly 2-hour sessions with an extended 6-hour class in the seventh week<br>**Type of delivery:** in-person group sessions	No intervention control group	T1 - baseline<br>T2 - post-intervention<br>T3 - 3 month follow up	CD-RISC 25	Yes	Yes	No	18 (fair)
Clarkson at al. (2019), UK	NRCS	During exposure	Pre-registration therapeutic radiography students	N=8<br>Male - 50%<br>Female - 50%<br>Mean age - 30	N=6<br>Male - 33,3%<br>Female - 66,7%<br>Mean age - 26	Aim: explore how mindfulness, as a self- care strategy, may influence resilience, compassion fatigue and burnout in pre-registration students.	Mindfulness-Based Resilience Training (Kabat-Zinn, 1990)	**Description:** During these sessions participants received training in four mindfulness practices: the Body Scan practice involves moving one’s attention progressively through the body observing physical sensations in each region; Mindful Movement, including gentle stretching, yoga based postures; Walking Meditation designed to enhance mindful awareness of the body in motion; Sitting Meditation, bringing mindfulness to the sensations of breathing, physical sensations,sounds, thoughts and emotions. Participants received compact disks recordings of all four practices to use daily for 30 min of home practice. Informal practices and reflection were introduced to emphasize bringing mindfulness to daily life.<br>**Duration of the program:** 5 weeks<br>**Duration of the sessions:** weekly 2 and half hours sessions<br>**Type of delivery:** in-person group sessions	No intervention control group	T1 - baseline<br>T2 - post-intervention<br>T3 - 3 month follow up<br>T4- 12 month follow up	CD-RISC 10	Yes	No	No	9 (poor)
Denkova et al. (2020), USA	NRCS	During exposure	Firefighters	Mindfulness Training:<br>N=42<br>Male - 83,33%<br>Female -16,67%<br>Mean age - 43.61; SD - 8.23<br><br>Relaxation training: N=31<br>Male - 80,65%<br>Female -19,35%<br>Mean age -45.38; SD - 6.80	N=48<br>Male - 79,17%<br>Female -20,83%<br>Mean age - 43.12; SD - 8.30	Aim: examine the impact of MT when compared to a well-matched, active-control training program (i.e., relaxation training, RT) on self-report measures of psychological resilience and affect, as well as on objective attentional task performance	Minfulness-Based Attention Training (MBAT);<br>Relaxation Training:<br>based on prior stress-reduction and relaxation practices that are traditionally utilized in evidence-based psychotherapies(e.g.,Cognitive Behavioral Therapy)	**Description:**<br>MT: Four central themes: concentration (focused attention, mind wandering); body awarness (body sensations and learning to observe the arising of impulses and distinguishing "over-reactions" from "responses"); open monitoring (self-regulation skills); connection (adaptative and effective leadership, team cohesion, kindness/connection practices)<br>RT: Didactic information, discussions, and practice with an emphasis on connecting the effects of a relaxed state to firefighter demands. Exercises were introduced corresponding to each central topic and included guided visualization, guided breathing, and progressive muscle relaxation.<br>All training participants were provided with MP3 devices that contained eight audio-guided exercises. The MT and RT programs were matched across all delivery structure dimensions (i.e.,number,length,and type of session as well as for the number and length of the out-of-class assignments). The sessions occurred over the same interval and were offered by the same training instructor.<br>**Duration of the program:** 4 weeks<br>**Duration of the sessions:** weekly 2-hour sessions; out-of-class formal practice assignments of 10–15 min of daily mindfulness and relaxation exercises, respectively<br>**Type of delivery:** in-person group sessions	No intervention control group	T1 - baseline<br>T2 - post-intervention	CD-RISC 25	No	No	MT - Yes<br>RT - No	14 (poor)
Erogul et al. (2014), USA	RCT	During exposure	Medical students	N=28<br>Male - 57,1%<br>Female - 42,9%<br>Mean age - 23.6; SD - 1.9	N=29<br>Male - 51,7%<br>Female - 48,3%<br>Mean age - 23.3; SD - 1.4	Aim: determine whether a variation of the standard MBSR program could help a randomized sample of 1st-year medical students in a lasting and sustained way compared to a control group	Mindfulness-Based Stress Reduction (MBSR)	**Description:** Experiential practices of mindfulness based meditation, body scan and breathing-based yoga to provide a cognitive curriculum about understanding stress and how best to manage reactivity. After week 4, the participants shifted from guided to self-meditation. The full-day retreat occurred offsite from 10 a.m. to 3 p.m.<br>**Duration of the program:** 8 weeks<br>**Duration of the sessions:** weekly 75 minutes session witth homework (daily meditation for 20-minutes); between the 7th and 8th weekly meeting, students attended a full-day retreat offsite<br>**Type of delivery:** in-person group sessions	No intervention control grup	T1 - baseline<br>T2 - post-intervention<br>T3 - 6 month follow-up	Resilience Scale	Yes	Yes	No	18 (fair)

Fikretoglu et al. (2019), Canada	RCT	During exposure	Military recruits	N=1239<br>Male - 86,53%<br>Female -13,47%<br>Mean age - 23.47; SD - 5.51	N=1083<br>Male - 84,48%<br>Female -15,52%<br>Mean age - 23.38; SD - 5.13	Aim: 1) to increase mental health literacy, 2) to teach stress management skills, and 3) to change attitudes and intentions towards mental health service use<br>Hypothesis: R2MR would have a beneficial effect on individual-level i) psychological functioning, ii) resilience, iii) MHSU attitudes, intentions, and behaviours, and iv) military performance	Mental Health Continuum Model	**Description:** Didactic modules on four skills: tactical (diaphragmatic) breathing, goal-setting, visualization, and self-talk. After this, participants are given hypothetical vignettes to help further reinforce mental health literacy and stress management skills.<br>**Duration of the program:** approximately 7 weeks<br>**Duration of the sessions:** 160 minute sessions<br>**Type of delivery:** in-person group session	Delayed intervention control group	T1 - baseline (2 days before exposure of IG)<br>T2 - during<br>T3 - post-intervention (2 days before exposure of CG)	CD-RISC 10	Yes	No	No	24 (good)
Giovannetti et al. (2020); Italy	RCT	Durng exposure	Adults with multiple sclerosis	N=18<br>Male - 28%<br>Female -72%<br>Mean age - 44.8; SD - 10.1	N=19<br>Male - 53%<br>Female - 47%<br>Mean age - 46.53; SD - 8.3	Aim: assess the efficacy of the Italian READY for MS program when compared to a active control intervention (group relaxation)	Cognitive Behaviour Therapy (CBT); Acceptance and Commitment Therapy (ACT)	**Description:** Content of the sessions is as follows: an introductory module, five modules focusing on each of the six ACT processes (Mindfulness, Acceptance, Cognitive Defusion, Self-as-Context, Values and Meaningful Action), and a review module (Review and Future Planning). The booster session provides a review of the program content. The program has a facilitator manual, participant workbook, and audio recordings of mindfulness exercises.<br>**Duration of the program:** 12 weeks<br>**Duration of the sessions:** seven weekly 2 and a half hour sessions plus a 2 and a half hour 'booster' session approximately five weeks after the seventh session; with homework<br>**Type of delivery:** in-person group sessions	Attention control group<br>**Description:** Relaxation program based on autogenic training.<br>**Duration of the sessions:** seven, 1-hour weekly sessions, followed by a 'booster' session after five weeks	T1 - baseline<br>T2 - post-intervention<br>T3 - after booster session<br>T4 - 3 month follow-up	Italian version of CD-RISC 25	Yes	Yes	No	20 (good)
Hendriks et al. (2019), Suriname	RCT	Pre-exposure	Suriname workers	N=80<br>Male - 47,5%<br>Female -52,5%<br>Mean age - 36.32; SD - 9.64	N=78<br>Male - 32.1%<br>Female -67.9%<br>Mean age - 36.11; SD - 9.43	Aim: examine the efficacy of the program, which was specifically designed to increase resilience;<br>Hypothesis: the Strong Minds Suriname program would significantly increase resilience and wellbeing among workers in Suriname compared to wait-list control.	Shell Resilience Program; Cognitive Behavioral Therapy; Positive Pshychology	**Description:** Divided in 5 modules - be grateful (relaxation, breathing, writing, verbal expression); positivity (relaxation, breathing, emotions, kindness); strenghts (relaxation, breathing, verbal expression, pshycho-education); goals (pshycho-education, physical exercise, verbal expression); overcoming problems (pshycho-education, writing,); let it go (pshycho-education, awarness, forgivness, gratitude through prayer, verbal expression)<br>**Duration of the program:** 6 weeks<br>**Duration of the sessions:** weekly 2 to 3 hour sessions, some with optional homework<br>**Type of delivery:** in-person group session (with individual exercises)	Wait-list control group	T1 - baselne<br>T2 - post-intervention<br>T3 - 3 month follow-up	Dutch Resilience Scale	Yes	Yes	Yes	20 (good)
Houston et al. (2017), USA	RCT	During exposure	College students	N=64	N=65	Aim: evaluate the efficacy of the program<br>Hypothesis: participants assigned to the RCI condition will report an increase in resilience, coping, and hope and a decrease in stress, depression, and anxiety compared with the control group	Cognitive Behvaiour Therapy (CBT)	**Description:** Focus on a specific problem that is shared by the group. The problem to be discussed in an RCI session can be identified by facilitators before a session (eg, a recent community disaster) or by participants as part of the session - facilitator leads the group through several steps to describe the problem, explore thoughts and feelings related to it, identify new problems, brainstorms options for change and to develop an individual and group action plan (coping strategies, connects, problem solving, satisfaction to help others, express feelings and thoughts).<br>**Duration of the program:** 3 weeks<br>**Duration of the sessions:** weekly 45 minute sessions<br>**Type of delivery:** in-person group session	No intervention control group	T1 - baseline<br>T2 - post-intervention	CD-RISC 25	Yes	Yes	Yes	14 (poor)
Hsieh et al. (2020), China	RCT	Pos- exposure	Abused Psychiatric Nurses	BT: N=49<br>Male - 12, 24%<br>Female - 87.76%<br>Mean age - 38.45; SD - 9.23<br><br>SDBT: N=47<br>Male - 38,3%<br>Female - 61.70%<br>Mean age -32.21 ;SD - 6.36	N=39<br>Male - 2,57%<br>Female -97,43%<br>Mean age - 35.61; SD - 7.47	Aim: compare the efectiveness of a BT and a smartphone-delivered BT (SDBT) intervention on the occupational stress, depressive symptoms, resilience, HRV, and respiration rate	Biofeedback Meditation	**Description:**<br>BT - Self-guided muscle relaxation, diaphragmatic breathing, paced breathing, pursed- ips breathing, and real-time respiratory sinus arrhythmia (RSA) biofeedback and shorter meditation practices.<br>SDBT - MP4 video file containing guided shorter meditation practices and processes of real-time biofeedback.<br>**Duration of the program:** 6 weeks<br>**Duration of the sessions:**<br>BT - weekly 60 minute sessions<br>SDBT - weekly self training<br>**Type of delivery:**<br>BT - in-person<br>SDBT - smartphone delivered	No intervention control group	T1 - baseline<br>T2 - post-intervention	Chinese version of Resilience Scale	Yes	Yes	BT - Yes<br>SDBT - Yes	15 (fair)
Hwang et al. (2017), Korea	RCT	Pre-exposure	Healthy office workers and graduate students	N=33<br>Male - 21,21%<br>Female -78,79%<br>Mean age - 30.82; SD - 4.82	N=18<br>Male - 33,33%<br>Female -66,67%<br>Mean age - 31.22; SD - 5.14	Aim: examine the effects of a short but intensive mindfulness meditation training program (Templestay program) and relaxation on individuals' resilience	Buddhism; Meditation; Relaxation	**Description:** Living at the temple, buddhist service, meditation, saring thoughts and life plans, farewells with monk, walk, write meditation log.<br>**Duration of the program:** 3 days and 4 nights<br>**Duration of the sessions:** retreat with 12 sessions totalling 19 hours of meditation practices<br>**Type of delivery:**in-person group sessions	Attention control group<br>**Description:** Living at the temple, not include set activities, merely rested.	T1 - baseline<br>T2 - post-intervention<br>T3 - 3 month follow-up	Resilience Quotient Test	No	No	Yes	13 (poor)
Kim et al. (2018), Korea	RCT	During exposure	Employees	VC: N=18<br>Male - 5,6%<br>Female - 94,4%<br>Mean age - 36.2; SD - 9.2<br><br>IP: N=27<br>Male - 7,4%<br>Female - 92,6%<br>Mean age -36.7; SD - 10.3	N=27<br>Male - 7,4%<br>Female - 92,6%<br>Mean age - 46.6; SD - 9.6	Aim: compare the effects of mobile videoconference-based intervention on stress reduction and resilience enhancement with that of in-person and self-care methods in Korean employees.	Stress Managment and Resilience Training: Relaxation Response Resilience Program (SMART-3RP)	**Description:**<br>IP - Understanding stress and relaxation response, creating adaptive perspectives through correctionof cognitive distortions, promoting positivity through a healthy lifestyle, humor, empathy and staying resilient.<br>VC - "Hello Mindcare" Android app that provides mobile counselling services, booking system, videoconferencing, document sharing and workbooks for clients to fill in directly.<br>**Duration of the program:** 4 weeks<br>**Duration of the sessions:** weekly 50 minute session<br>**Type of delivery:**<br>VC - smartphone app<br>IP -in-person individual sessions	Attention control group<br>**Description:** received educational material regarding methods to self-regulate stress.<br>**Duration of the sessions:** were instucted to read one chapter weekly	T1 - baseline<br>T2 - post-intervention<br>T3 - 1 month follow-up	Brief Resilience Scale	No	Yes	Yes	20 (good)
Kiropoulos et al. (2016), Australia	RCT	During exposure	Depressed individuals newly diagnosed with multiple sclerosis	N=15<br>Male - 13,3%<br>Female -86,7%<br>Mean age - 34.60; SD - 9.06	N=15<br>Male - 40%<br>Female -60%<br>Mean age - 39.27; SD - 9.93	Aim: assess the efficacy of an intervention in the treatment of depressive symptoms (primary outcome) in individuals who are within five years of a MS diagnosis; secondary aims were to examine improvements in levels of anxiety, fatigue, pain, sleep quality, quality of life, coping, MS illness acceptance and resilience	Cognitive Behavioral Therapy (CBT)	**Description:** Progressive muscle relaxation, controlled breathing exercises, pleasant activity scheduling, problem solving skills, cognitive exercises which helped individuals identify, challenge and manage unhelpful thoughts and beliefs; focused on facilitating acceptance of MS illness and adjusting to living with MS and the final therapy session was dedicated to relapse prevention.<br>**Duration of the program:** 8 weeks<br>**Duration of the sessions:** 1-hour session (apart from the first that was 1 and half hours)<br>**Type of delivery:** in-person individual session	Usual care control group<br>**Description:** did not obtain any psychological treatment for depressive symptoms or anxiety for the entire length of their involvement in the trial and received usual medical care from their neurologist which may include 1-3 visits for medication review.	T1- baseline<br>T2 - post-intervention<br>T3 - 20-week follow-up	Resilience Scale for Adults	Yes	No	Yes	19 (fair)
Kovacs et al. (2018), North America	RCT	During exposure	Adults with Congenital Heart Disease	N=14<br>Male - 48%<br>Female -52%<br>Mean age - 33.1; SD - 11.2	N=12<br>Male - 52%<br>Female -48%<br>Mean age - 32.5; SD - 12.5	Aim: report the feasibility of a intervention aimed at improving psychosocial functioning, quality of life (QOL), and resilience	Cognitive Behavioral Therapy (CBT); Relaxation training	**Description:** Education about living with CHD (eg, prevalence and common psychosocial challenges), cognitive-behavioural coping strategies and social interaction and communication skills training through relaxation training (diaphragmatic breathing, progressive muscle relaxation, autogenic training, and guided imagery), cognitive restructuring, behavioural activation (planning pleasant events), physical activity, activity pacing, and strategies to improve sleep and assertive communication. Participants were provided a binder and weekly handouts.<br>**Duration of the program:** 8 weeks<br>**Duration of the sessions:** weekly 90 minute sessions; homework<br>**Type of delivery:** in-person group sessions	Usual care control group	T1 - baseline<br>T2 - post-intervention<br>T3 - 3 month follow-up	Resilience Scale	Yes	No	No	19 (fair)

Lee et al. (2020), Korea	NRCS	During exposure	Hospital workers	N=25<br>Male - 0%<br>Female -100%<br>Mean age - 36.20; SD - 8.17	N=31<br>Male - 0%<br>Female -100%<br>Mean age - 35; SD - 6.74	Aim: evaluate the effects of an online program on participants' stress, anger, coping strategies, emotional intelligence, resilience and positive and negative affect	Mind-body training (MBT)	**Description:** Exercises of brain relaxation, breathing techinques, meditation, stretching and others divided in 10 phases that are repeated four times over the course.<br>**Duration of the program:** 8 weeks<br>**Duration of the sessions:** once per day, 5 days per week, with each daily session lasting 10 minutes<br>**Type of delivery:** online	No intervention control group	T1 - baseline<br>T2 - during<br>T3 - post-intervention<br>T4 - 1 month follow-up	Korean version of CD-RISC 25	No	Yes	Yes	15 (fair)
Lin et al. (2018), China	RCT	During exposure	Nurses	N=44<br>Male - 2,3%<br>Female -97,7%<br>Mean age - 32.86; SD - 7.49	N=46<br>Male - 10,9%<br>Female -89,1%<br>Mean age - 30.20; SD - 6.09	Aim: evaluate the effects of a modified mindfulness-based stress reduction (MBSR) program on the levels of stress, affect, and resilience	Mindfulness-Based Stress Reduction (MBSR); Mindfulness-Based Cognitive Therapy (MBCT) (Kabat-Zinn, 1990)	**Description:** Guided practice, education, and dialogues around participants' observations of feelings, thoughts, and body sensations during practice. In addition, a network Chatgroup through WeChat (Tencent Inc., China) on mobile phones, sending session PowerPoint slides and audio recordings of guided mindfulness exercises, which helped the participants to share their practice experience or to ask the MBSR instructor questions.<br>**Duration of the program:** 8 weeks<br>**Duration of the sessions:** weekly 2-hour sessions, 20 minutes of formal mindfulness practice for six days a week<br>**Type of delivery:** in-person group sessions	No intervention control group	T1 - baseline<br>T2 - post-intervention<br>T3 - 3 month follow-up	Chinese version of CD-RISC 25	No	Yes	Yes	14 (poor)
Loprinzi et al. (2011), USA	RCT	Pos- exposure	Breast cancer survivors	N=12<br>Male - 0%<br>Female -100%<br>Median age - 61	N=8<br>Male - 0%<br>Female -100%<br>Median age - 61	Aim: test a Stress Management and Resiliency Training (SMART) program for enhancing resilience and well-being and for decreasing stress and anxiety	Attention and Interpretation Therapy (AIT)	**Description:** Paced breathing meditation, attention training (exercises to help patients direct their interpretations away from fixed prejudices and toward a more flexible disposition while cultivating skills such as gratitude, compassion, acceptance, forgiveness, and higher meaning and purpose).<br>**Duration of the program:** 12 weeks<br>**Duration of the sessions:** 2 small-group sessions of 90 minutes each, brief (30-60 minute) optional individual session and 3 follow-up (4-week intervals) telephone calls<br>**Type of delivery:** in-person group and individual sessions and telephone calls	Wait-list control group	T1 - baseline<br>T2 - post-intervention	CD-RISC 25	Yes	Yes	Yes	17 (fair)
Mache et al. (2015), Germany	RCT	During exposure	Junior Physicians	N=42<br>Male - 38%<br>Female -62%<br>Mean age - 28	N=43<br>Male - 41%<br>Female -59%<br>Mean age - 28	Aim: gather preliminary information regarding the feasibility of implementing a psychosocial resilience program and to assess if the program would potentially promote protective factors (such as resiliency, self efficacy and job satisfaction as well as decreasing perceived stress	Cognitive Behavioral Therapy (CBT); solution-focused counseling	**Description:** Session focused in instructing and promoting fundamental communication, goal-setting, improving emotional problems, increasing motivation, self-efficacy, etc through watching videos, discussions, experiential exercises, etc.<br>**Duration of the program:** 3 months<br>**Duration of the sessions:** weekly 2-hour sessions; homework<br>**Type of delivery:** in-person group sessions	No intervention control group	T1 - baseline<br>T2 - post-intervention<br>T3 - 6 month follow-up	German version of the Brief Resilience Scale	No	Yes	Yes	18 (fair)
McCann, Songprakun & Stephenson (2016) , Thailand	RCT	During exposure	Primary caregivers of family members with depression	N=27<br>Male - 55.6%<br>Female -44,4%<br>Mean age - 41; SD - 8.2	N=27<br>Male - 40,7%<br>Female -59.3%<br>Mean age - 41; SD - 9.8	Aim: assess the efficacy of a GSH manual in increasing resilience in family caregivers of individuals diagnosed with moderate depression in comparison to caregivers who only received routine outpatient department support	Cognitive Behaviour Therapy (CBT); Guided self-help (GSH)	**Description:** Participants received The Good Mood Guide: A Self-Help Manual for Depression, which was translated into Thai and comprised eight modules: (i) gives an outline of depression and encourages readers to engage in physical activity; (ii) affirms the importance of maintaining social contact and physical exercise; (iii) enables participants to discern the way they feel and think; (iv) emphasizes how to change thought patterns from negative to positive; (v) highlights how healthy living, social support, and problem solving enable behaviour change and contribute to overcoming depression; (vi) equips the person to improve sleep pattern and to sustain favourable thoughts, behaviours, and emotions; (vii) illustrates how to practise progressive muscle relaxation to assist them deal with stress; and (viii) reemphasizes earlier learned skills in thought challenging, dealing with difficult events and changing behaviour. Received weekly telephone calls from a researcher to give limited support and to anwser questions about using the manual.<br>**Duration of the program:** 12 weeks<br>**Duration of the sessions:** one module per week (each took approximately 2 hours to complete) and short (approximately 5 minutes) weekly telephone call from a researcher.<br>**Type of delivery:** self-admnistrated and telephone calls	Usual care control group<br>**Description:** standard support while accompanying the family member with depression to the outpatient department for prescription of antidepressant or anti-anxiety and antidepressant medications and consultations. Standard support included receiving minimal support and information from mental health nurses about supporting the affected family member.Also received short (approximately 5 minutes) weekly telephone calls from a researcher to give limited support.	T1 - baseline<br>T2 - post-intervention<br>T3 - 1 month follow-up	Thai version of the Resilience Scale	No	No	Yes	20 (good)
McGonagle, Beatty & Joffe (2014), USA	RCT	During exposure	Full-time working adults with chronic health conditions	N=23<br>Mean age - 38.30; SD - 8.2<br>Predominately female (86%),	N=25<br>Mean age - 39.07; SD - 7.79<br>Predominately female (86%),	Aim: examine if the coaching intervention would have positive effects on individual workers' perceived work ability, job satisfaction, resilience and other personal resources and would help decrease burnout levels	Resource activation drawing upon two theories of stress: the transactional model and conservation of resources (COR) theory; GROW coaching model	**Description:** Set goals, create awarness, self-assessment, identification of possible solutions, develop action plans. Homeworks include creating task lists and refleting on them or behavioral routines, reading, journaling or meditation.<br>**Duration of the program:** 12 weeks<br>**Duration of the sessions:** six 1-hour coaching sessions (one session every other week); homework<br>**Type of delivery:** telephone calls	Wait-list control group	T1 - baseline<br>T2 - pos-intervention<br>T3 - 3 month follow-up	CD-RISC 10	Yes	Yes	Yes	16 (fair)
Mealer et al. (2014), USA	RCT	During exposure	ICU nurses	N=13<br>Male - 8%<br>Female - 92%	N=14<br>Male - 14%<br>Female - 86%	Aim: determine if a mulitmodal resilience training program for ICU nurses was feasible and acceptable and to identify an effect size of the intervention and the prevalence of psychological disorders	Cognitive Behaviour Therapy (CBT); Pennerbaker's expressive writing framework; Mindfulness-Based Stress Reduction (MBSR)	**Description:** Two-day Educational Workshop, guided mindfulness sessions and provided guided compact discs for use, expressive writing, stress-reduction techinques based on mindfulness (body scan), exercise, event-triggered counseling sessions.<br>**Duration of the program:** 12 weeks<br>**Duration of the sessions:** 2-day educational worshop with 2-hour guided mindfulness exercise sessions and 4-hour introduction to expressive writing; twelve 30 minute sessions of expressive writing; practice MBSR techiques as a homework for 15 minutes at least 3 times per week during 1 weeks; 3 month membership to the welness center was provided for participants to engage in 30 to 45 minutes of aerobic exercise at least 3 days per week; counseling sessions (number depending) of 30 to 60 minutes<br>**Type of delivery:** telephone calls	No intervention control group	T1 - baseline<br>T2 - pos-intervention	CD-RISC 25	Yes	No	No	18 (fair)
Mondanaro et al. (2020), USA	RCT	During exposure	Cancer patients doing infusion therapy	N=43<br>Male - 23%<br>Female -77%<br>Mean age - 55; SD - 13.05	N=44<br>Male - 20%<br>Female -80%<br>Mean age - 53.41; SD - 11.94	Aim: investigate the effectiveness of clinical music improvisation on the resilience of adults undergoing infusion therapy	Music Therapy	**Description:**<br><u>VI -</u> Participated in 5 minutes of warm up selected and sang a song or several favorite; engaged in song writing, or improvised with expanded singing of 10 minutes or more, inclusive of clinical improvisation (melody, harmony, timbre, and rhythmic idioms) where musical communication was fostered and therapeutic goals were addressed by the therapist within the music; identified themes and issues addressed and/or paralleled in the song and/or within the self; and obtained closure through verbal reflection and sharing (five minutes).<br><u>II -</u> Participated in 5 minutes of warm up after selecting an instrument of choice (and instruments for caregiver/s if applicable); played during expanded playing of 10 minutes or more; the rest is the same as VI.<br>**Duration of the program:** 1 to 3 months<br>**Duration of the sessions:** 3 sessions of 20 minutes each upon enrollement, midpoint and completion of the chemotherapy regimen<br>**Type of delivery:** in-person individual session	No intervention control group	T1 - baseline<br>T2 - pos-intervention	Resilience Scale	Yes	No	Yes	15 (fair)
Pidgeon, Ford & Klaassen (2013), Australia	RCT	During exposure	Human service professionals	N=16<br>Male - 9%<br>Female - 91%<br>Mean age - 40.7; SD - 12.28	N=14<br>Male - 9%<br>Female - 91%<br>Mean age - 40.7; SD - 12.28	Aim: evaluate the efficacy of a Mindfulness with Metta Training Programme (MMTP) as a pathway to foster resilience in a sample of human services professionals	Mindfulness, loving-kindness meditation, and cognitive therapy	**Description:** Periods of silence, training in mindfulness, metta skills and cognitive therapy strategies to increase mindfulness and self-compassion. Booster sessions including a review of the retreat. A meditation CD was provided.<br>**Duration of the program:** 4 months<br>**Duration of the sessions:** two and a half day retreat; booster sessions of 4 hours at one and four months<br>**Type of delivery:** in-person group session	Wait-list control group	T1 - baseline<br>T2 - post-intervention<br>T23 - 1 month follow-up<br>T4 - 4 month follow-up	Resilience Scale	Yes	No	Yes	13 (poor)

Roig et al. (2020), Ireland	RCT	During exposure	College students	With human support:<br>N=29<br>Male - 21%<br>Female -79%<br><br>With automated support:<br>N=26<br>Male - 15%<br>Female -85%	N=28<br>Male - 15%<br>Female -85%	Aim: investigate the feasibility of a new web-based resilience program, provided with human or automated support, in a sample of college students	Positive psychology and cognitive behavorial elements	**Description:**<br>HS: Supporters were counsellors or trainee counsellors familiar with using the SilverCloud Health platform. The role of the supporter was to monitor and support user progress through the program. Using this information, supporters spent 10-15 minutes formulating individualized reviews for each participant. Participants received 4 reviews during the intervention period.<br>AS: Received generic, templated reviews which were automatically sent as messages on the platform. Automated reviews were predeveloped by highly experienced clinicians with in-depth knowledge of providing support for web-based interventions. Participants received 4 reviews during the intervention period.<br>Space for Resilience is a 7-module program that includes introductory videos, quizzes, psychoeducational content, personal stories from other users, interactive activities, mindfulness exercises, homework suggestions, goal setting, and summaries.<br>**Duration of the program:** 8 weeks<br>**Duration of the sessions:** accessible 24/7 - it was recommended that participants spend atleast an hour a week on the program<br>**Type of delivery:** self-administered through an internet program	Wait-list control group	T1 - baseline<br>T2 - post-intervention	CD-RISC 25; Brief Resilience Scale	Yes	Yes	HS: Yes<br>AS: Yes	18 (fair)
Senders et al. (2019), USA	RCT	During exposure	Adults with multiple sclerosis	N=33<br>Male - 15,15%<br>Female - 84,85%<br>Mean age - 53.24; SD - 10.66	N=29<br>Male - 31,3%<br>Female - 68,97%<br>Mean age - 52.59; SD - 12.31	Aim: evaluate efficacy of MBSR compared to an education control	Mindfulness-based Stress Reduction (MBSR)	**Description:** Bring mindfulness into daily practice through meditation, movement, eating, and interacting with others. Instruction included a variety of techniques to facilitate this practice, including gentle yoga, breath work, and body scan. Participants were encouraged to practice 45 minutes daily.<br>**Duration of the program:** 8 weeks<br>**Duration of the sessions:** weekly 2-hour classes and a 6-hour retreat during week six; homework<br>**Type of delivery:** in-person group session	Attention control group<br>**Description:** The program was facilitated by a Program Implementation and Engagement Coordinator from the National MS Society Oregon Chapter. Classes were structured around pamphlets published by the National MS Society, with topics that included: medications and supplements, fatigue, pain, gait and balance, cognition and mood, knowing your rights, financial planning, and connecting with resources. During the retreat participants watched three brief documentaries about stress, mood, and self-efficacy and engaged in facilitated discussion.	T1 - baseline<br>T2 - during (week 4)<br>T3 - post-intervention<br>T4 - 4 month follow-up<br>T5 - 8 month follow-up<br>T6 - 12 month follow-up	CD-RISC 25	Yes	Yes	No	20 (good)
Songprakun & McCann (2012), Thailand	RCT	During exposure	Adults with depression	N=26<br>Male - 18,5%<br>Female - 81,5%<br>Mean age - 39.4; SD - 10.1	N=28<br>Male - 34,5%<br>Female - 65,5%<br>Mean age - 44.7; SD - 8.8	Aim: examine the effectiveness of a self-help manual on the resilience levels of individuals with depression	Cognitive Behaviour Therapy (CBT); Guided self-help (GSH)	**Description:** Use of a self-help manual and workbook (Good Mood Guide: A self-help manual for depression). The manual had eight modules (one module was completed each week) which contained principles and activities to be completed each week including reading, questionnaires and homework exercises which individuals were encouraged to undertake between sessions in order to challenge unhelpful thoughts and behaviours and to strengthen their resilience. Intervention group participants could use the programme to help them control negative emotions and encourage them to engage in daily living activities. Received weekly telephone calls from a researcher to give limited support and to awnser questions about using the manual. Also received standard care and treatment.<br>**Duration of the program:** 8 weeks<br>**Duration of the sessions**: one module per week (each took approximately 2 hours to complete) and short (approximately 5 minutes) weekly telephone call from a researcher<br>**Type of delivery:** self-admnistrated and telephone calls	Attention control group<br>**Description:** standard care and treatment.Also received short (approximately 5 minutes) weekly telephone calls from a researcher to give limited support.	T1 - baseline<br>T2 - post-intervention<br>T3 - 1 month follow-up	Resilience Scale	No	No	Yes	21 (good)
Sood et al. (2011), USA	RCT	During exposure	Physicians	N=20<br>Male - 55%<br>Female - 45%<br>Mean age - 46.8; SD - 8.3	N=12<br>Male - 50%<br>Female - 50%<br>Mean age - 50.2; SD - 5.7	Aim: assess the effect of a Stress Management and Resiliency Trainin (SMART) program for increasing resiliency and quality of life, and decreasing stress and anxiety among physicians	Attention and Interpretation Therapy (AIT)	**Description:** Techniques like paced breathing meditation, attention training (exercises to help patients direct their interpretations away from fixed prejudices and toward a more flexible disposition while cultivating skills such as gratitude, compassion, acceptance, forgiveness, and higher meaning).<br>**Duration of the program:** 8 weeks<br>**Duration of the sessions**: single 90 minute session with an optional 30 to 60 minute follow-up session depending on individual needs<br>**Type of delivery:** in-person individual session	Wait-list control group	T1 - baseline<br>T2 - post-intervention	CD-RISC 25	Yes	Yes	Yes	16 (fair)
Sood et al. (2014), USA	RCT	During exposure	Physicians	N=13<br>Male - 55%<br>Female - 45%<br>Mean age - 47.4; SD - 8.8	N=13<br>Male - 50%<br>Female - 50%<br>Mean age - 48.1; SD - 5.2	Aim: test the efficacy of a Stress Management and Resiliency Training (SMART) program for decreasing stress and anxiety and improving resilience and quality of life among physicians	Attention and Interpretation Therapy (AIT)	**Description:** Techniques like attention training (exercises to help patients direct their interpretations away from fixed prejudices and toward a more flexible disposition while cultivating skills such as gratitude, compassion, acceptance, forgiveness, and higher meaning), paced breathing meditation. Participants were provided reading materials that covered skills discussed and were offered and optional follow-up session and 2 follow-up phone calls.<br>**Duration of the program:** 12 weeks<br>**Duration of the sessions**: single 90 minute session with an optional 30 to 60 minute follow-up session depending on individual needs and 2 follow-up phone calls at weeks 4 and 8<br>**Type of delivery:** in-person group session and telephone calls	Wait-list control group	T1 - baseline<br>T2 - post-intervention	CD-RISC 25	No	Yes	No	18 (fair)
van Grieken et al. (2019), Netherlands	NRCS	During exposure	Parents at risk of parenting problems (opportunity sample)	N=124<br>Male - 10%<br>Female -90%<br>Mean age - 31.0; SD - 7	N=177<br>Male - 7%<br>Female -93%<br>Mean age - 30.7; SD - 5.3	Aim: assess efects of the program on on parenting skills, social support (both from family and friends, and from the partner), self-sufficiency and resilience	Ecological model of Belsky and Newberger's concept of parental awarness which was elaborated upon by Baartman	**Description:** Activating social networks, increasing parenting skills and supporting parent(s)/caregiver(s) in getting grip of their life; empowering and worrisome experiences are adressed<br>**Duration of the program:** first 18 months after childbirth<br>**Duration of the sessions:** six 90 minute sessions (usually every 3 months)<br>**Type of delivery:** in-person individual sessions (home visits)	Usual care control group<br>**Description:** provided by the Youth Health Care Centers consisting on regular well-child check-ups at set ages (12 check-ups in the first 18 months after childbirth) common advice regarding parenting; if needed parents can be referred to specialized professional care.<br>**Duration of the sessions:** 20 minutes sessions	T1 - baseline (12 months of child age)<br>T2 - post-intervention (18 months of child age)	Dutch Resilience Scale	No	No	No	19 (fair)
Weiss et al. (2020), Netherlands	RCT	During exposure	Vulnerable adults	N=58<br>Male - 27,6%<br>Female - 73,4%<br>Median age - 59	N=50<br>Male - 34%<br>Female - 66%<br>Median age - 61	Aim: examine effectiveness effects of the intervention in comparison to Customized Care regarding the (a) primary outcome measure of well-being and the (b) secondary outcome measures of resilience, purpose in life, depression, health-related quality of life, loneliness, social participation and health care costs	Positive psychology using principles of self-determination theory	**Description:** Techniques like examining values, behavioural activation or life-review methods, examine future dreams, awarness of passions (choose an activity), skills development. The participants were allowed to spend up to 500 euros to realize their passion. The money could be used to pay for an activity (e.g., a painting course or yoga classes) or to purchase something needed for an activity (e.g., a camera or a season ticket to the local football club).<br>**Duration of the program:** 3 months<br>**Duration of the sessions**: 2 to 6 home visits with intervals of generally between 1 to 3 weeks; the sessions had a maximum duration of 90 minutes<br>**Type of delivery:** in-person individual session	Attention control group<br>**Description:** Provided best possible care through the traditional problem-focus approach with the objective of controlling for nonspecific treatment effects.<br>**Duration of the sessions**: 2 home visits with a maximum duration of 90 minutes	T1 - baseline<br>T2 - post-intervention<br>T3 - 9 month follow-up	Brief Resilience Scale	Yes	No	No	19 (fair)
Wesner et al. (2019), Brasil	RCT	During exposure	Adults with panic disorder	N=50<br>Male - 14%<br>Female - 86%<br>Mean age - 36.8; SD - 11.5	N=50<br>Male - 22%<br>Female - 78%<br>Mean age - 38.1; SD - 11	Aim: evaluating the efficacy of four booster sessions focusing on resilience and coping strategies added to the standard CBGT protocol for panic disorder	Cognitive Behavioral Group Therapy (CBGT)	**Description:** Used elements like coping strategies, emotion-focused strategies (use of adaptative resources and mood modulation, problem-solving, decision-making, communication and interpersonal negotiation skills), adaptative thoughts, promoting resilience.<br>**Duration of the program:** 4 months<br>**Duration of the sessions:** sixteen 90 minute sessions<br>**Type of delivery**: in-person group sessions	Usual care control group<br>**Description:** Used elements like psychoeducation, anxiety management techniques, cognitive restructuring, interoceptive and naturalistic exposure, and finally *in vivo* exposure.<br>**Duration of the sessions:** twelve 90 minute sessions (8 weekly and 4 every other week)	T1 - baseline<br>T2 - post-intervention	Brazilian Portuguese version of the Resilience Scale	Yes	No	Yes	18 (fair)

Wild et al. (2020), England	RCT	During exposure	Emergency workers	N=317<br>Male - 41.32%<br>Female - 58.68%<br>Mean age - 41.09; SD - 9.98	N=113<br>Male - 43.36%<br>Female - 56.64%<br>Mean age - 42.32; SD - 9.20	Aim: evaluate the effectiveness of a tertiary service resilience intervention compared to psychoeducation for improving psychological outcomes among emergency workers	Blue Light programme by Mind	**Description:** Experiential exercises drawn from stress management and mindfulness, with the overarching aim to improve wellbeing and use of adaptive coping strategies, such as social support.<br>**Duration of the program:** 6 weeks<br>**Duration of the sessions:** weekly 2 and half hour sessions; homework<br>**Type of delivery**: in-person group sessions	Attention control group<br>**Description:** Psychoeducation about six topics: sleep, stress, depression, anger, mindfulness, and post-traumatic stress disorder.<br>**Duration of the sessions:** release of one module per week<br>**Type of delivery**: online	T1 - baseline<br>T2 - post-intervention<br>T3 - 3 month follow-up	CD-RISC 10	Yes	No	No	20 (good)
Wong et al. (2019), Hong-Kong	RCT	During exposure	Female sex workers	N=63<br>Female - 100%<br>Mean age - 42.1; SD - 6.6	N=64<br>Female -100%<br>Mean age - 38.2; SD - 6.7	Aim: investigate the effectiveness and explore a mediation model of a resilience intervention in improving both the psychological and sexual health of FSWs	Transactional Model of Stress and coping and resilience framework; Pshychoeducation, cognitive-behavioural strategies and social learning principles	**Description:** Games, role play, brainstorming, case scenarios and discussions.<br>**Duration of the program:** between 15 and 18 weeks<br>**Duration of the sessions:** six 1-hour sessions with 1-3 week intervals<br>**Type of delivery**: in-person group sessions	Usual care control group<br>**Description:** Provided by the NGO's including outreach visits offering HIV/STI testing and social activities.	T1 - baseline<br>T2 - post-intervention<br>T3 - 3 month follow-up	CD-RISC 25	Yes	No	Yes	17 (fair)
Wu et al. (2018), Taiwan	RCT	During exposure	Paients with breast cancer under chemotherapy	N=20<br>Female - 100%<br>Mean age - 51.2; SD - 9.18	N=20<br>Female - 100%<br>Mean age - 51.2; SD - 10.71	Aim: evaluate the effects of the<br>PEI in addressing anxiety, depression, disease-specific care knowledge, quality of life, self-efficacy, and resilience in patients with breast cancer undergoing chemotherapy.	Transactional Model of Stress and coping	**Description:** Sessions took place during five chemotherapy treatments. The broad scope of the PEI was divided into two categories: (a) an educational manual that offered information and activities related to depression, anxiety, disease-specific care knowledge, self-efficacy, and resilience and (b) a self-assessment of learning. Activities including relaxation techniques, experience sharing, adaptation techniques, emotional management, scenarios and forums.<br>**Duration of the program:** between 15 and 18 weeks<br>**Duration of the sessions:** six 1-hour sessions<br>**Type of delivery**: in-person group sessions	Usual care control group	T1 - baseline<br>T2 - during (3rd session)<br>T3 -during (5th session)<br>T4 - post-intervention (2 weeks after)	Resilience Scale	Yes	No	Yes	17 (fair)
Ye et al. (2017), China	RCT	Pos- exposure	Metastatic breast cancer survivors	N=94<br>Female - 100%	N=89<br>Female - 100%	Hypothesis: BRBC would (a) prolong 3- and 5-year survival, (b) decrease emotional distress (anxiety, depression, pain and so on) andphysical distress (allostatic load), and (c) increase the resilience and QoL.	Supportive-expressive group therapy (SEGT)	**Description:** Foster self-efficacy to combat symptoms (such as pain, fatigue, intrusive thoughts and so on) through knowledge and techniques (such as breath control, meditation and so on), and to help patients gain a sense of control in their life.<br>**Duration of the program:** 12 months<br>**Duration of the sessions:** weekly 120 minutes sessions (45 minutes for education part)<br>**Type of delivery**: in-person group sessions	Attention control group<br>**Description:** Received a CD containing relaxation therapy (developed by the authors) was provided to every CG participant and monthly telephone follow-up was performed to prevent demoralisation from random assignment.	T1 - baseline<br>T2 - during (2 months)<br>T3 -during (6 months)<br>T4 - post-intervention	CD-RISC 10	Yes	No	Yes	21 (good)
Yu et al. (2014), Hong Kong	RCT	During exposure	Chinese new immigrants to Hong Kong	IA: N=63<br>Male - 3,2%<br>Female - 96,8%<br>Mean age - 31.92; SD - 4.61<br><br>RA: N=58<br>Male - 7,2%<br>Female - 94,8%<br>Mean age -32.97; SD - 4.46	N=62<br>Male - 4,8%<br>Female - 95,2%<br>Mean age - 33.84; SD - 5.56	Aim: test the effectiveness of two interventions used to decrease adaptation difficulties by (a) providing knowledge of resources that are relevant to the Hong Kong context or (b) enhancing personal resilience in immigrants	Community-based participatory research model	**Description:**<br>IA - didactic sessions aimed at increasin participants' knowledge about education, medical care, housing, employment, and community resources.<br>RA - self-efficacy, positive thinking, altruism (volunteering), goal setting.<br>**Duration of the program:** 12 months<br>**Duration of the sessions:**<br>IA - two 2 and half hour sessions<br>RA - four weekly 2 and half hour sessions<br>**Type of delivery**: in-person group sessions	Attention control group<br>**Description:** Received a 16-page informational booklet relevant to education, medical care, housing, employment, and community resources	T1 - baseline<br>T2 - post-intervention<br>T3 - 3 month follow-up	CD-RISC 25	Yes	No	IA: Yes<br>RA: Yes	21 (good)
